# Global existence and blow-up results for *p*-Laplacian parabolic problems under nonlinear boundary conditions

**DOI:** 10.1186/s13660-018-1665-3

**Published:** 2018-04-03

**Authors:** Juntang Ding

**Affiliations:** School of Mathematical Sciences, Shanxi University, Taiyuan, P.R. China

**Keywords:** 35K65, 35B40, Blow-up, *p*-Laplacian equation, Nonlinear boundary condition

## Abstract

This paper is devoted to studying the global existence and blow-up results for the following *p*-Laplacian parabolic problems:

$$\textstyle\begin{cases} (h(u) )_{t} =\nabla\cdot (|\nabla u|^{p-2}\nabla u )+f(u) &\mbox{in } D\times(0,t^{*}), \\ \frac{\partial u}{\partial n}=g(u) &\mbox{on } \partial D\times (0,t^{*}), \\ u(x,0)=u_{0}(x)\geq0 & \mbox{in } \overline{D}. \end{cases} $$

Here $p>2$, the spatial region *D* in $\mathbb{R}^{N}$ ($N\geq2$) is bounded, and *∂D* is smooth. We set up conditions to ensure that the solution must be a global solution or blows up in some finite time. Moreover, we dedicate upper estimates of the global solution and the blow-up rate. An upper bound for the blow-up time is also specified. Our research relies mainly on constructing some auxiliary functions and using the parabolic maximum principles and the differential inequality technique.

## Introduction

For more than ten years, many authors have discussed the blow-up phenomena of *p*-Laplacian parabolic problems. We refer the readers to [1–11] and the references therein. In this paper, we intend to study the blow-up phenomena of the following *p*-Laplacian parabolic problems:

1.1 $$ \textstyle\begin{cases} (h(u) )_{t} =\nabla\cdot (|\nabla u|^{p-2}\nabla u )+f(u) &\mbox{ in } D\times(0,t^{*}), \\ \frac{\partial u}{\partial n}=g(u) &\mbox{ on } \partial D\times (0,t^{*}), \\ u(x,0)=u_{0}(x)\geq0 & \mbox{ in } \overline{D}. \end{cases} $$

In (1.1), $p>2$, the spatial region *D* in $\mathbb{R}^{N}$ ($N\geq2$) is bounded, *∂D* is smooth, $t^{*}$ is the blow-up time if the blow-up occurs, otherwise $t^{*}=+\infty$, $h(s)$ is a $C^{2}(\overline{\mathbb{R}_{+}})$ function with $h'(s)>0$, $s\in \overline{\mathbb{R}_{+}}$, $f(s)$ is a positive $C^{1}(\overline {\mathbb{R}_{+}})$ function, $g(s)$ is a positive $C^{2}(\overline{\mathbb{R}_{+}})$ function, and $u_{0}(x)$ is a nonnegative $C^{2}(\overline{D})$ function with $\partial u_{0}(x)/\partial n=g(u_{0})$, $x\in\partial D$. The above assumptions and the regularity theorem in [12] guarantee that the nonnegative classical solution *u* of problem (1.1) satisfies $u\in C^{3}({D}\times(0,T))\cap C^{2}({\overline{D}}\times[0,T))$.

The blow-up problems for parabolic equations with nonlinear boundary conditions have been widely investigated in recent years (see, e.g., [1, 6, 13–26]). In order to study problem (1.1), we focus on the papers [1, 16], and [22]. In [1], Ding and Shen considered the following problems:

1.2 $$ \textstyle\begin{cases} (h(u) )_{t} =\nabla\cdot (|\nabla u|^{p-2}\nabla u )+f(u) &\mbox{in } D\times(0,t^{*}), \\ |\nabla u|^{p-2}\frac{\partial u}{\partial n}=g(u) &\mbox{on } \partial D\times(0,t^{*}), \\ u(x,0)=u_{0}(x)\geq0 & \mbox{in } \overline{D}. \end{cases} $$

In (1.2), $p\geq2$, the spatial region *D* in $\mathbb{R}^{N}$ ($N\geq2$) is bounded and convex, and *∂D* is smooth. By constructing some auxiliary functions and using the differential inequality technique, they established the conditions on functions *f*, *g*, *h*, and $u_{0}$ to ensure that the solution *u* blows up at some time. In addition, an upper bound and a lower bound of the blow-up time were obtained. The method in [1] is not suitable for the study of problem (1.1) because of the different boundary conditions of problem (1.1) and problem (1.2). Zhang et al. [16] and Zhang [22] dealt with the following problem:

1.3 $$ \textstyle\begin{cases} (h(u) )_{t} =\nabla\cdot(a(u)\nabla u)+f(u) &\mbox{in } D\times(0,t^{*}), \\ \frac{\partial u}{\partial n}=g(u) &\mbox{on } \partial D\times (0,t^{*}), \\ u(x,0)=u_{0}(x)>0 & \mbox{in } \overline{D}. \end{cases} $$

In (1.3), the spatial region *D* in $\mathbb{R}^{N}$ ($N\geq2$) is bounded, and *∂D* is smooth. By constructing some auxiliary functions and using parabolic maximum principles, they set up the conditions on functions *a*, *f*, *g*, *h*, and $u_{0}$ to guarantee that the solution either blows up in a finite time or exists globally. Moreover, an upper estimate of the blow-up rate and an upper bound of the blow-up time are given. They also obtained an upper estimate of the global solution. We intend to use the methods in [16] and [22] to study problem (1.1). Since the principal parts of the two equations are different in problems (1.1) and (1.3), the auxiliary functions in papers [16] and [22] are not suitable for problem (1.1). Therefore, the key to our research is to construct some new auxiliary functions. By using these new auxiliary functions, parabolic maximum principles, and differential inequality techniques, we complete the study of problem (1.1).

We proceed as follows. In Sect. 2, we set up some conditions to ensure that the solution blows up in a finite time. An upper estimate of the blow-up solution and an upper bound of the blow-up time are also given. Section 3 is devoted to finding some conditions to guarantee that the solution exists globally. At the same time, we also obtain an upper estimate of the global solution. In Sect. 4, as applications of the abstract results, two examples are presented.

In this paper, for convenience, we use a comma to denote partial differentiation, for example, $u_{,i}=\frac{\partial u}{\partial x_{i}}$, $u_{,ij}=\frac{\partial^{2}u}{\partial x_{i}\, \partial x_{j}}$. We also adopt summation convection, for example,

$$u_{,i}u_{,j}u_{,ij}=\sum ^{n}_{i=1}\sum^{n}_{j=1} \frac{\partial u}{\partial x_{i}}\frac{\partial u}{\partial x_{j}}\frac{\partial^{2}u}{ \partial x_{i}\, \partial x_{j}}. $$

## Blow-up solution

In order to study the blow-up solution of (1.1), we define

2.1 $$ \alpha=\inf_{s\in\overline{\mathbb{R}_{+}}}\frac {f(s)}{g(s)h'(s){\mathrm{e}}^{-s}} $$

and

2.2 $$ \beta=\min_{\overline{D}} \frac{\nabla\cdot (|\nabla u_{0}|^{p-2}\nabla u_{0} )+f(u_{0})}{ g(u_{0})h'(u_{0}){\mathrm{e}}^{-u_{0}}}. $$

We also construct the following two auxiliary functions:

2.3 $$\begin{aligned}& P(x,t)=-\frac{1}{g(u)}u_{t}+\beta{ \mathrm{e}}^{-u}, \quad (x,t)\in\overline {D}\times \bigl[0,t^{*} \bigr), \end{aligned}$$

2.4 $$\begin{aligned}& \Phi(s)= \int^{+\infty}_{s}\frac{{\mathrm{e}}^{\tau}}{g(\tau)}\, {\mathrm{d}}\tau, \quad s\in\overline{\mathbb{R}_{+}}. \end{aligned}$$

Since $g(s)$ is a positive $C^{2}(\overline{\mathbb{R}_{+}})$ function, we have

$$\Phi'(s)=-\frac{{\mathrm{e}}^{s}}{g(s)}< 0, \quad s\in\mathbb{R}_{+}, $$

which means that the function Φ has an inverse function $\Phi ^{-1}$. With the aid of the above two auxiliary functions, we can get the following Theorem 2.1 that is the main result on the blow-up solution.

### Theorem 2.1

*Let* *u* *be a nonnegative classical solution of* (1.1). *Assume that the following three assumptions are true*:

(i) 2.5 $$ 0< \beta\leq\alpha; $$
(ii) 2.6 $$ \int^{+\infty}_{M_{0}}\frac{{\mathrm{e}}^{\tau}}{g(\tau)}\, {\mathrm{d}}\tau < + \infty, \qquad M_{0}=\max_{\overline{D}}u_{0}(x); $$
(iii) 2.7 $$ \begin{aligned} &1-2\frac{g'(s)}{g(s)}+ \frac{g''(s)}{g(s)}\geq0, \qquad (p-2) \biggl(\frac{g'(s)}{g(s)}-1 \biggr)- \frac{h''(s)}{h'(s)}\geq 0, \\ &\frac{f'(s)}{f(s)}-(p-1) \biggl(\frac{g'(s)}{g(s)}-1 \biggr)\geq0, \quad s\in \overline{\mathbb{R}_{+}}. \end{aligned} $$

*Then the solution* *u* *must blow up in a finite time* $t^{*}$ *and*

$$t^{*}\leq\frac{1}{\beta} \int^{+\infty}_{M_{0}}\frac{{\mathrm{e}}^{\tau }}{g(\tau)}\, {\mathrm{d}}\tau $$

*as well as*

$$u(x,t)\leq\Phi^{-1} \bigl(\beta \bigl(t^{*}-t \bigr) \bigr), \quad (x,t) \in \overline{D}\times\bigl[0,t^{*}\bigr). $$

### Proof

For the auxiliary function $P(x,t)$ defined in (2.3), by calculating, we have

2.8 $$ P_{,i}=\frac{g'}{g^{2}}u_{t}u,_{i}- \frac{1}{g}u_{t{,}i}-\beta{\mathrm{e}}^{-u}u_{,i} $$

and

2.9 $$\begin{aligned} P_{,ij} =& \biggl(\frac{g''}{g^{2}}-2\frac{(g')^{2}}{g^{3}} \biggr)u_{t}u_{,i}u_{,j}+\frac{g'}{g^{2}}u_{,i}u_{t,j} +\frac{g'}{g^{2}}u_{t}u_{,ij}+\frac{g'}{g^{2}}u_{t,i}u_{,j}- \frac{1}{g}u_{t{,}ij} \\ &{}+\beta{\mathrm{e}}^{-u}u_{,i}u_{,j}-\beta{ \mathrm{e}}^{-u}u_{,ij}. \end{aligned}$$

With (2.9), we get

2.10 $$\begin{aligned} \Delta P =&P_{,ii} \\ =& \biggl(\frac{g''}{g^{2}}-2\frac{(g')^{2}}{g^{3}} \biggr)|\nabla u|^{2}u_{t} +2\frac{g'}{g^{2}} (\nabla u\cdot\nabla u_{t} ) +\frac{g'}{g^{2}}u_{t}\Delta u-\frac{1}{g} \Delta u_{t} \\ &{}+\beta{\mathrm{e}}^{-u}|\nabla u|^{2} -\beta{ \mathrm{e}}^{-u}\Delta u. \end{aligned}$$

Using the first equation of (1.1), we obtain

2.11 $$\begin{aligned} P_{t} =&\frac{g'}{g^{2}} (u_{t} )^{2}- \frac{1}{g} (u_{t} )_{t}-\beta{\mathrm{e}}^{-u}u_{t} \\ =&\frac{g'}{g^{2}} (u_{t} )^{2}-\beta{ \mathrm{e}}^{-u}u_{t} -\frac{1}{g} \biggl( \frac{\nabla\cdot (|\nabla u|^{p-2}\nabla u )}{h'}+\frac{f}{h'} \biggr)_{t} \\ =&\frac{g'}{g^{2}} (u_{t} )^{2}-\beta{ \mathrm{e}}^{-u}u_{t}+\frac {h''}{g(h')^{2}}|\nabla u|^{p-2}u_{t}\Delta u -(p-2)\frac{1}{gh'}|\nabla u|^{p-4} (\nabla u\cdot\nabla u_{t} )\Delta u \\ &{}-\frac{1}{gh'}|\nabla u|^{p-2}\Delta u_{t}+(p-2) \frac{h''}{g(h')^{2}}|\nabla u|^{p-4}u_{t}u_{,i}u_{,j}u_{,ij} \\ &{}-(p-2) (p-4)\frac{1}{gh'}|\nabla u|^{p-6} (\nabla u\cdot\nabla u_{t} )u_{,i}u_{,j}{u_{,ij}} - {2(p-2)} \frac{1}{gh'}|\nabla u|^{p-4}u_{t,i}u_{,j}u_{,ij} \\ &{}-(p-2)\frac{1}{gh'}|\nabla u|^{p-4}u_{,i}u_{,j}u_{t,ij} + \biggl(\frac{fh''}{g(h')^{2}}-\frac{f'}{gh'} \biggr)u_{t}. \end{aligned}$$

It follows from (2.9)–(2.11) that

2.12 $$\begin{aligned}& \frac{1}{h'}|\nabla u|^{p-2}\Delta P+(p-2)\frac{1}{h'}| \nabla u|^{p-4}u_{,i}u_{,j}P_{,ij}-P_{t} \\& \quad = (p-1) \biggl(\frac{g''}{g^{2}h'}-2\frac{(g')^{2}}{g^{3}h'} \biggr)|\nabla u|^{p}u_{t} +2(p-1)\frac{g'}{g^{2}h'}|\nabla u|^{p-2} (\nabla u\cdot\nabla u_{t} ) \\& \qquad {} + \biggl(\frac{g'}{g^{2}h'}-\frac{h''}{g(h')^{2}} \biggr)|\nabla u|^{p-2}u_{t}\Delta u +\beta(p-1)\frac{{\mathrm{e}}^{-u}}{h'}|\nabla u|^{p} -\beta\frac{{\mathrm{e}}^{-u}}{h'}|\nabla u|^{p-2}\Delta u \\& \qquad {} +(p-2) \biggl(\frac{g'}{g^{2}h'}-\frac{h''}{g(h')^{2}} \biggr)|\nabla u|^{p-4}u_{t}u_{,i}u_{,j}u_{,ij} -\beta(p-2)\frac{{\mathrm{e}}^{-u}}{h'}|\nabla u|^{p-4}u_{,i}u_{,j}u_{,ij} \\& \qquad {} -\frac{g'}{g^{2}}(u_{t})^{2} + \biggl(\beta{ \mathrm{e}}^{-u}+\frac{f'}{gh'}-\frac {fh''}{g(h')^{2}} \biggr)u_{t} +(p-2)\frac{1}{gh'}|\nabla u|^{p-4} (\nabla u \cdot\nabla u_{t} )\Delta u \\& \qquad {} +(p-2) (p-4)\frac{1}{gh'}|\nabla u|^{p-6} (\nabla u\cdot \nabla u_{t} )u_{,i}u_{,j}u_{,ij} \\& \qquad {}+2(p-2)\frac{1}{gh'}|\nabla u|^{p-4}u_{t},_{i}u_{,j}u_{,ij}. \end{aligned}$$

By (2.8), we have

2.13 $$ u_{t,i}=-gP_{,i}+\frac{g'}{g^{2}}u_{t}u_{,i}- \beta g{\mathrm{e}}^{-u}u_{,i} $$

and

2.14 $$ \nabla u_{t}=-g\nabla P+\frac{g'}{g}u_{t} \nabla u-\beta g{\mathrm{e}}^{-u}\nabla u. $$

Inserting (2.13) and (2.14) into (2.12), we arrive at

2.15 $$\begin{aligned}& \frac{1}{h'}|\nabla u|^{p-2}\Delta P+(p-2)\frac{1}{h'}| \nabla u|^{p-4}u_{,i}u_{,j}P_{,ij} \\& \qquad {}+\frac{1}{h'}|\nabla u|^{p-6} \biggl(2(p-1) \frac{g'}{g}|\nabla u|^{4}+(p-2) (p-4)u_{,i}u_{,j}u_{,ij} +(p-2)|\nabla u|^{2}\Delta u \biggr) \\& \qquad {}\times (\nabla u\cdot\nabla P )+2(p-2)\frac{1}{h'}|\nabla u|^{p-4}u_{,j}u_{,ij}P_{,i}-P_{t} \\& \quad = (p-1)\frac{g''}{g^{2}h'}|\nabla u|^{p}u_{t} + \biggl(\beta(p-1)\frac{{\mathrm{e}}^{-u}}{h'}-2\beta(p-1)\frac{g'{\mathrm{e}}^{-u}}{gh'} \biggr)|\nabla u|^{p} \\& \qquad {} + \biggl((p-1)\frac{g'}{g^{2}h'}-\frac{h''}{g(h')^{2}} \biggr)|\nabla u|^{p-2}u_{t}\Delta u -\beta(p-1)\frac{{\mathrm{e}}^{-u}}{h'}|\nabla u|^{p-2}\Delta u \\& \qquad {} +(p-2) \biggl((p-1)\frac{g'}{g^{2}h'}-\frac{h''}{g(h')^{2}} \biggr)| \nabla u|^{p-4}u_{t}u_{,i}u_{,j}u_{,ij} \\& \qquad {}-\beta(p-1) (p-2)\frac{{\mathrm{e}}^{-u}}{h'}|\nabla u|^{p-4}u_{,i}u_{,j}u_{,ij} \\& \qquad {} -\frac{g'}{g^{2}} (u_{t} )^{2} + \biggl(\beta{ \mathrm{e}}^{-u}+\frac{f'}{gh'}-\frac{fh''}{g(h')^{2}} \biggr)u_{t}. \end{aligned}$$

It follows from the first equation of (1.1) that

2.16 $$ |\nabla u|^{p-2}\Delta u=h'u_{t}-(p-2)| \nabla u|^{p-4}u_{,i}u_{,j}u_{,ij}-f. $$

Substituting (2.16) into (2.15), we deduce

2.17 $$\begin{aligned}& \frac{1}{h'}|\nabla u|^{p-2}\Delta P+(p-2)\frac{1}{h'}| \nabla u|^{p-4}u_{,i}u_{,j}P_{,ij} \\& \qquad {} +\frac{1}{h'}|\nabla u|^{p-6} \biggl(2(p-1) \frac{g'}{g}|\nabla u|^{4}+(p-2) (p-4)u_{,i}u_{,j}u_{,ij} +(p-2)|\nabla u|^{2}\Delta u \biggr) \\& \qquad {} \times (\nabla u\cdot\nabla P ) +2(p-2)\frac{1}{h'}|\nabla u|^{p-4}u_{,j}u_{,ij}P_{,i}-P_{t} \\& \quad = (p-1)\frac{g''}{g^{2}h'}|\nabla u|^{p}u_{t} + \biggl(\beta(p-1)\frac{{\mathrm{e}}^{-u}}{h'}-2\beta(p-1)\frac{g'{\mathrm{e}}^{-u}}{gh'} \biggr)|\nabla u|^{p} \\& \qquad {} + \biggl((p-2)\frac{g'}{g^{2}}-\frac {h''}{gh'} \biggr) (u_{t})^{2}+ \biggl(\frac{f'}{gh'}-(p-1) \frac{g'f}{g^{2}h'}-\beta(p-2){\mathrm{e}}^{-u} \biggr)u_{t} \\& \qquad {}+\beta(p-1)\frac{f{\mathrm{e}}^{-u}}{h'}. \end{aligned}$$

With (2.3), we have

2.18 $$ u_{t}=gP+\beta g{\mathrm{e}}^{-u}. $$

Inserting (2.18) into (2.17), we derive

2.19 $$\begin{aligned}& \frac{1}{h'}|\nabla u|^{p-2}\Delta P+(p-2)\frac{1}{h'}| \nabla u|^{p-4}u_{,i}u_{,j}P_{,ij} \\& \qquad {} +\frac{1}{h'}|\nabla u|^{p-6} \biggl(2(p-1) \frac{g'}{g}|\nabla u|^{4}+(p-2) (p-4)u_{,i}u_{,j}u_{,ij} +(p-2)|\nabla u|^{2}\Delta u \biggr) \\& \qquad {} \times (\nabla u\cdot\nabla P ) +2(p-2)\frac{1}{h'}|\nabla u|^{p-4}u_{,j}u_{,ij}P_{,i} \\& \qquad {} + \biggl[(p-1)\frac{g''}{gh'}|\nabla u|^{p} + \biggl( \frac{gh''}{h'}-(p-2)g' \biggr) \bigl(P-2\beta{ \mathrm{e}}^{-u} \bigr) \\& \qquad {}+\frac{f'}{h'}-(p-1)\frac{g'f}{gh'}-\beta(p-2)g{ \mathrm{e}}^{-u} \biggr]P-P_{t} \\& \quad = \beta(p-1)\frac{{\mathrm{e}}^{-u}}{h'} \biggl(1-2\frac{g'}{g}+ \frac {g''}{g} \biggr)|\nabla u|^{p} +\beta^{2}g{ \mathrm{e}}^{-2u} \biggl[(p-2) \biggl(\frac{g'}{g}-1 \biggr)- \frac {h''}{h'} \biggr] \\& \qquad{} +\beta\frac{f{\mathrm{e}}^{-u}}{h'} \biggl[\frac{f'}{f}-(p-1) \biggl( \frac{g'}{g}-1 \biggr) \biggr]. \end{aligned}$$

Assumption (2.7) guarantees that the right-hand side of equality (2.19) is nonnegative. Hence, we have

2.20 $$\begin{aligned}& \frac{1}{h'}|\nabla u|^{p-2}\Delta P+(p-2) \frac{1}{h'}| \nabla u|^{p-4}u_{,i}u_{,j}P_{,ij} \\& \quad {}+\frac{1}{h'}|\nabla u|^{p-6} \biggl(2(p-1) \frac{g'}{g}| \nabla u|^{4}+(p-2) (p-4)u_{,i}u_{,j}u_{,ij} +(p-2)|\nabla u|^{2}\Delta u \biggr) \\& \quad {} \times (\nabla u\cdot\nabla P ) +2(p-2)\frac{1}{h'}|\nabla u|^{p-4}u_{,j}u_{,ij}P_{,i} \\& \quad {} + \biggl[(p-1)\frac{g''}{gh'}|\nabla u|^{p} + \biggl( \frac{gh''}{h'}-(p-2)g' \biggr) \bigl(p-2\beta{ \mathrm{e}}^{-u} \bigr) \\& \quad {}+\frac{f}{h'}-(p-1)\frac{g'f}{gh'}- \beta(p-2)g{ \mathrm{e}}^{-u} \biggr]P \\& \quad {} -P_{t}\geq0 \quad \mbox{in } D\times \bigl(0,t^{*} \bigr). \end{aligned}$$

The regularity assumptions on functions *f*, *g*, and *h* in Sect. 1, parabolic maximum principles [27], and (2.20) imply that under the following three possible cases, *P* may take its nonnegative maximum value:

- for $t=0$,
- at a point where $|\nabla u|=0$,
- on the boundary $\partial D\times(0,t^{*})$.

First, we consider case (a). With (2.2), we deduce

2.21 $$\begin{aligned} P(x,0) =&-\frac{1}{g(u_{0})} \biggl\{ \frac{1}{h'(u_{0})} \bigl[ \nabla \cdot \bigl(|\nabla u_{0}|^{p-2}\nabla u_{0} \bigr) +f(u_{0}) \bigr] \biggr\} +\beta{\mathrm{e}}^{-u_{0}} \\ =&{\mathrm{e}}^{-u_{0}} \biggl(\beta-\frac{\nabla\cdot (|\nabla u_{0}|^{p-2}\nabla u_{0} )+f(u_{0})}{ g(u_{0})h'(u_{0}){\mathrm{e}}^{-u_{0}}} \biggr) \\ \leq&0\quad \mbox{in } \overline{D}. \end{aligned}$$

Then, we consider case (b). Assume that $(\tilde{x},\tilde{t})\in D\times(0,t^{*})$ is a point where $|\nabla u(\tilde{x},\tilde{t})|=0$. Now we have

$$\begin{aligned} \bigl\vert \nabla\cdot \bigl(|\nabla u|^{p-2}\nabla u \bigr) \bigr\vert =& \bigl\vert |\nabla u|^{p-2}\Delta u+(p-2)|\nabla u|^{p-4}u_{,i}u_{,j}u_{,ij} \bigr\vert \\ \leq&|\nabla u|^{p-2}|\Delta u|+(p-2)|\nabla u|^{p-4}|\nabla u||\nabla u||u_{,ij}| \\ =&|\nabla u|^{p-2} \bigl(|\Delta u|+(p-2)|u_{,ij}| \bigr). \end{aligned}$$

Hence, we obtain

$$\bigl\vert \nabla\cdot \bigl(|\nabla u|^{p-2}\nabla u \bigr) \bigr\vert \big| _{(\tilde{x},\tilde{t})} \leq\frac{}{}|\nabla u|^{p-2} \bigl(|\Delta u|+(p-2)|u_{,ij}| \bigr) \big| _{(\tilde{x},\tilde{t})}=0; $$

that is,

2.22 $$ \frac{}{}\nabla\cdot \bigl(|\nabla u|^{p-2} \nabla u \bigr) \big| _{(\tilde{x},\tilde{t})}=0. $$

It follows from (2.22), (2.1), and (2.5) that

2.23 $$\begin{aligned} P(\tilde{x},\tilde{t}) =& \biggl(-\frac{1}{g(u)}u_{t}+ \beta{ \mathrm{e}}^{-u} \biggr) \bigg| _{(\tilde{x},\tilde{t})} = \biggl\{ - \frac{1}{g(u)h'(u)} \bigl[\nabla\cdot \bigl(|\nabla u|^{p-2}\nabla u_{0} \bigr)+f(u) \bigr] +\beta{\mathrm{e}}^{-u} \biggr\} \bigg|_{(\tilde{x},\tilde{t})} \\ =& \biggl(-\frac{f(u)}{g(u)h'(u)}+\beta{\mathrm{e}}^{-u} \biggr) \bigg|_{(\tilde{x},\tilde{t})} = \biggl[{\mathrm{e}}^{-u} \biggl(\beta- \frac{f(u)}{g(u)h'(u){\mathrm{e}}^{-u}} \biggr) \biggr] \bigg| _{(\tilde{x},\tilde{t})} \\ \leq&\frac{}{}{\mathrm{e}}^{-u}(\beta-\alpha) \big| _{(\tilde {x},\tilde{t})} \leq0. \end{aligned}$$

Finally, we consider case (c). Making use of the boundary condition of (1.1), we get

2.24 $$\begin{aligned} \frac{\partial P}{\partial n} =& \frac{g'}{g^{2}}u_{t} \frac{\partial u}{\partial n}- \frac{1}{g}\frac {\partial u_{t}}{\partial n} -\beta{ \mathrm{e}}^{-u} \frac{\partial u}{\partial n} =\frac{g'}{g}u_{t}- \frac{1}{g} \biggl( \frac{\partial u}{\partial n} \biggr)_{t}-\beta{ \mathrm{e}}^{-u}g \\ =&\frac{g'}{g}u_{t}-\frac{1}{g}g_{t}-\beta{ \mathrm{e}}^{-u}g =-\beta{\mathrm{e}}^{-u}g< 0 \quad \mbox{on } \partial D\times \bigl(0,t^{*} \bigr). \end{aligned}$$

Parabolic maximum principles, (2.21), and (2.23)–(2.24) guarantee that the maximum value of *P* in $\overline {D}\times[0,t^{*})$ is nonpositive. Hence, we have

$$P(x,t)\leq0 \quad \mbox{in } \overline{D}\times\bigl[0,t^{*}\bigr), $$

from which we obtain the following differential inequality:

2.25 $$ \frac{{\mathrm{e}}^{u}}{\beta g(u)}u_{t}\geq1. $$

At the point $\bar{x}\in\overline{D}$, where $u_{0}(\bar{x})=M_{0}$, integrating (2.25) from 0 to *t*, we derive

2.26 $$ \frac{1}{\beta} \int^{t}_{0}\frac{{\mathrm{e}}^{u}}{g(u)}u_{t}\, { \mathrm{d}}t =\frac{1}{\beta} \int^{u(\bar{x},t)}_{M_{0}}\frac{{\mathrm{e}}^{\tau }}{g(\tau)}\, {\mathrm{d}}\tau \geq t, $$

which implies that *u* must blow up in a finite time $t^{*}$. In fact, suppose that *u* is a global solution, then for any $t>0$, we deduce

2.27 $$ \frac{1}{\beta} \int^{+\infty}_{M_{0}}\frac{{\mathrm{e}}^{\tau}}{g(\tau )}\, {\mathrm{d}}\tau > \frac{1}{\beta} \int^{u(\bar{x},t)}_{M_{0}}\frac{{\mathrm{e}}^{\tau }}{g(\tau)}\, {\mathrm{d}}\tau \geq t. $$

Taking the limit as $t\rightarrow+\infty$ in (2.27), we arrive at

$$\frac{1}{\beta} \int^{+\infty}_{M_{0}}\frac{{\mathrm{e}}^{\tau}}{g(\tau )}\, {\mathrm{d}}\tau=+ \infty, $$

which contradicts (2.6). This contradiction suggests that *u* must blow up in a finite time $t^{*}$. Letting $t\rightarrow t^{*}$ in (2.26), we have

$$t^{*}\leq\frac{1}{\beta} \int^{+\infty}_{M_{0}}\frac{{\mathrm{e}}^{\tau }}{g(\tau)}\, {\mathrm{d}}\tau. $$

For each fixed $x\in\overline{D}$, integrating (2.25) from *t* to *t̃* ($0< t<\tilde{t}<t^{*}$), we get

2.28 $$ \Phi \bigl(u(x,t) \bigr)\geq\Phi \bigl(u(x,t) \bigr)-\Phi \bigl(u(x, \tilde{t}) \bigr) = \int^{u(x,\tilde{t})}_{u(x,t)}\frac{{\mathrm{e}}^{\tau}}{g(\tau)}\, {\mathrm{d}}\tau \geq \beta(\tilde{t}-t). $$

Passing to the limit as $\tilde{t}\rightarrow t^{*}$ in (2.28), we obtain

$$\Phi \bigl(u(x,t) \bigr)\geq\beta \bigl(t^{*}-t \bigr), $$

from which we deduce

$$u(x,t)\leq\Phi^{-1} \bigl(\beta \bigl(t^{*}-t \bigr) \bigr). $$

The proof is complete. □

## Global solution

In order to complete the study of the global solution to (1.1), we define

3.1 $$ \xi=\sup_{s\in\overline{\mathbb{R}_{+}}}\frac{f(s)}{g(s)h'(s){\mathrm{e}}^{s}} $$

and

3.2 $$ \eta=\max_{\overline{D}}\frac{\nabla\cdot (|\nabla u_{0}|^{p-2}\nabla u_{0} )+f(u_{0})}{ g(u_{0})h'(u_{0}){\mathrm{e}}^{u_{0}}}. $$

We also construct the following two auxiliary functions:

3.3 $$\begin{aligned}& Q(x,t)=-\frac{1}{g(u)}u_{t}+\eta{ \mathrm{e}}^{u}, \quad (x,t)\in\overline {D}\times\bigl[0,t^{*}\bigr), \end{aligned}$$

3.4 $$\begin{aligned}& \Psi(s)= \int^{s}_{m_{0}}\frac{{\mathrm{e}}^{-\tau}}{g(\tau)}\, {\mathrm{d}}\tau, \quad s \geq m_{0}=\min_{\overline{D}}u_{0}(x). \end{aligned}$$

Here $g(s)$ is a positive $C^{2}(\overline{\mathbb{R}_{+}})$ function to ensure

$$\Psi'(s)=\frac{{\mathrm{e}}^{-s}}{g(s)}>0, \quad s\geq m_{0}. $$

This implies that the inverse function $\Psi^{-1}$ of the function Ψ exists. The following Theorem 3.1 is the main result of the global solution to problem (1.1).

### Theorem 3.1

*Let* *u* *be a nonnegative classical solution of* (1.1). *Assume that the following three assumptions are satisfied*:

(i) 3.5 $$ \eta\geq\xi>0; $$
(ii) 3.6 $$ \int^{+\infty}_{m_{0}}\frac{{\mathrm{e}}^{-\tau}}{g(\tau)}\, {\mathrm{d}}\tau =+ \infty; $$
(iii) 3.7 $$ \begin{aligned} &1+2\frac{g'(s)}{g(s)}+ \frac{g''(s)}{g(s)}\leq0, \qquad (p-2) \biggl(\frac{g'(s)}{g(s)}+1 \biggr)- \frac{h''(s)}{h'(s)}\leq0, \\ &\frac{f'(s)}{f(s)}-(p-1) \biggl(\frac{g'(s)}{g(s)}+1 \biggr)\leq0, \quad s\in \overline{\mathbb{R}}. \end{aligned} $$

*Then* *u* *must be a global solution and*

$$u(x,t)\leq\Psi^{-1} \bigl(\eta t+\Psi \bigl(u_{0}(x,t) \bigr) \bigr),\quad (x,t)\in\overline{D}\times\overline{\mathbb{R}^{+}}. $$

### Proof

By using the reasoning process (2.8)–(2.19) for the auxiliary function *Q* defined in (3.3), we have

3.8 $$\begin{aligned}& \frac{1}{h'}|\nabla u|^{p-2}\Delta Q+(p-2) \frac{1}{h'}| \nabla u|^{p-4}u_{,i}u_{,j}Q_{,ij} \\& \qquad {} +\frac{1}{h'}|\nabla u|^{p-6} \biggl(2(p-1) \frac{g'}{g}| \nabla u|^{4}+(p-2) (p-4)u_{,i}u_{,j}u_{,ij} +(p-2)|\nabla u|^{2}\Delta u \biggr) \\& \qquad {}\times (\nabla u\cdot\nabla Q )+2(p-2)\frac{1}{h'}|\nabla u|^{p-4}u_{,j}u_{,ij}Q_{,i} \\& \qquad {} + \biggl[(p-1)\frac{g''}{gh'}|\nabla u|^{p} + \biggl( \frac{gh''}{h'}-(p-2)g' \biggr) \bigl(Q-2\eta{ \mathrm{e}}^{u} \bigr) \\& \qquad {}+\frac{f'}{h'}-(p-1)\frac{g'f}{gh'}+ \eta(p-2)g{ \mathrm{e}}^{u} \biggr]Q-Q_{t} \\& \quad = \eta(p-1)\frac{{\mathrm{e}}^{u}}{h'} \biggl(1+2\frac{g'}{g}+ \frac {g''}{g} \biggr)|\nabla u|^{p} +\eta^{2}g{ \mathrm{e}}^{2u} \biggl[(p-2) \biggl(\frac{g'}{g}+1 \biggr)- \frac {h''}{h} \biggr] \\& \qquad {} +\eta\frac{f{\mathrm{e}}^{u}}{h'} \biggl[\frac{f'}{f}-(p-1) \biggl( \frac {g'}{g}+1 \biggr) \biggr]. \end{aligned}$$

It follows from (3.7) and (3.8) that

$$\begin{aligned}& \frac{1}{h'}|\nabla u|^{p-2}\Delta Q+(p-2)\frac{1}{h'}| \nabla u|^{p-4}u_{,i}u_{,j}Q_{,ij} \\& \quad {}+\frac{1}{h'}|\nabla u|^{p-6} \biggl(2(p-1) \frac{g'}{g}|\nabla u|^{4}+(p-2) (p-4)u_{,i}u_{,j}u_{,ij} +(p-2)|\nabla u|^{2}\Delta u \biggr) \\& \quad {}\times (\nabla u\cdot\nabla Q )+2(p-2)\frac{1}{h'}|\nabla u|^{p-4}u_{,j}u_{,ij}Q_{,i} \\& \quad {}+ \biggl[(p-1)\frac{g''}{gh'}|\nabla u|^{p} + \biggl( \frac{gh''}{h'}-(p-2)g' \biggr) \bigl(Q-2\eta{ \mathrm{e}}^{u} \bigr) \\& \quad {}+\frac{f'}{h'}-(p-1)\frac{g'f}{gh'}+ \eta(p-2)g{\mathrm{e}}^{u} \biggr]Q \\& \quad {}-Q_{t} \leq0 \quad \mbox{in } D\times\bigl(0,t^{*}\bigr). \end{aligned}$$

The parabolic maximum principle guarantees that in the following three possible cases, *Q* may take its nonpositive minimum value:

- for $t=0$,
- at a point where $|\nabla u|=0$,
- on the boundary $\partial D\times(0,t^{*})$.

First, case (a) is considered. By (3.2), we deduce

3.9 $$\begin{aligned} Q(x,0) =&-\frac{1}{g(u_{0})} \biggl\{ \frac{1}{h'(u_{0})} \bigl[ \nabla \cdot \bigl(|\nabla u_{0}|^{p-2}\nabla u_{0} \bigr)+f(u_{0}) \bigr] \biggr\} +\eta{\mathrm{e}}^{u_{0}} \\ =&{\mathrm{e}}^{u_{0}} \biggl(\eta-\frac{\nabla\cdot (|\nabla u_{0}|^{p-2}\nabla u_{0} )+f(u_{0})}{ g(u_{0})h'(u_{0}){\mathrm{e}}^{u_{0}}} \biggr)\geq0 \quad \mbox{in } \overline{D}. \end{aligned}$$

Then, case (b) is considered. Repeating the reasoning process of (2.23) and using (2.22), (3.1), and (3.5), we have

3.10 $$ Q(\tilde{x},\tilde{t})= \biggl[{\mathrm{e}}^{u} \biggl(\eta-\frac {f(u)}{g(u)h'(u){\mathrm{e}}^{u}} \biggr) \biggr] \bigg| _{(\tilde{x},\tilde{t})} \frac{}{}\geq{\mathrm{e}}^{u}(\eta-\xi) \big| _{(\tilde {x},\tilde{t})} \geq0, $$

where $(\tilde{x},\tilde{t})\in D\times(0,t^{*})$ is a point where $|\nabla u(\tilde{x},\tilde{t})|=0$. Finally, case (c) is considered. With the aid of the reasoning process in (2.24), it is easy to get

3.11 $$ \frac{\partial Q}{\partial n}=\eta{\mathrm{e}}^{u} \frac{\partial u}{\partial n}=\eta{\mathrm{e}}^{u}g>0\quad \mbox{in } \partial D \times \bigl(0,t^{*} \bigr). $$

Combining (3.9)–(3.11) and parabolic maximum principles, we can obtain that the minimum value of *Q* in $\overline {D}\times[0,t^{*})$ is nonnegative. In other words, we have

$$Q(x,t)\geq0 \quad \mbox{in } \overline{D}\times\bigl[0,t^{*}\bigr), $$

which implies that the following differential inequality holds:

3.12 $$ \frac{{\mathrm{e}}^{-u}}{\eta g(u)}u_{t}\leq1. $$

For each fixed $x\in\overline{D}$, integrating (3.12) from 0 to *t*, we deduce

3.13 $$ \frac{1}{\eta} \int^{t}_{0}\frac{{\mathrm{e}}^{-u}}{g(u)}u_{t}\, { \mathrm{d}}t =\frac{1}{\eta} \int^{u(x,t)}_{u_{0}(x)} \frac{{\mathrm{e}}^{-\tau}}{g(\tau)}\, {\mathrm{d}}\tau \leq t, $$

which guarantees that *u* must be a global solution. In fact, if we assume that *u* blows up at a finite time $t^{*}$, then the following conclusion holds:

$$\lim_{t\rightarrow t^{*-}}u(x,t)=+\infty. $$

Letting $t\rightarrow t^{*-}$ in (3.13), we have

$$\frac{1}{\eta} \int^{+\infty}_{u_{0}(x)}\frac{{\mathrm{e}}^{-\tau }}{g(\tau)}\, {\mathrm{d}}\tau \leq t^{*} $$

and

$$\frac{1}{\eta} \int^{+\infty}_{m_{0}} \frac{{\mathrm{e}}^{-\tau}}{g(\tau)}\, {\mathrm{d}}\tau = \frac{1}{\eta} \int^{u_{0}(x)}_{m_{0}}\frac{{\mathrm{e}}^{-\tau}}{g(\tau )}\, {\mathrm{d}}\tau + \frac{1}{\eta} \int^{+\infty}_{u_{0}(x)}\frac{{\mathrm{e}}^{-\tau }}{g(\tau)}\, {\mathrm{d}}\tau \leq \frac{1}{\eta} \int^{u_{0}(x)}_{m_{0}}\frac{{\mathrm{e}}^{-\tau }}{g(\tau)}\, {\mathrm{d}} \tau+t^{*}< +\infty, $$

which contradicts (3.6). This shows that *u* must be a global solution. It follows from (3.13) that

$$\int^{u(x,t)}_{u_{0}(x)}\frac{{\mathrm{e}}^{-\tau}}{g(\tau)}\, {\mathrm{d}}\tau = \int_{m_{0}}^{u(x,t)}\frac{{\mathrm{e}}^{-\tau}}{g(\tau)}\, {\mathrm{d}}\tau - \int^{u_{0}(x)}_{m_{0}}\frac{{\mathrm{e}}^{-\tau}}{g(\tau)}\, {\mathrm{d}}\tau = \Psi \bigl(u(x,t) \bigr)-\Psi \bigl(u_{0}(x) \bigr) \leq\eta t, $$

from which we get

$$u(x,t)\leq\Psi^{-1} \bigl(\eta t+\Psi \bigl(u_{0}(x) \bigr) \bigr). $$

The proof is complete. □

## Applications

In this section, we give two examples to illustrate the results of Theorems 2.1 and 3.1.

### Example 4.1

Let *u* be a nonnegative classical solution of the following problem:

$$\textstyle\begin{cases} (u{\mathrm{e}}^{u} )_{t} =\nabla\cdot (|\nabla u|^{2}\nabla u )+{\mathrm{e}}^{6u} & \mbox{in }D\times(0,t^{*}), \\ \frac{\partial u}{\partial n}=2{\mathrm{e}}^{2(u-1)} & \mbox{on }\partial D\times(0,t^{*}), \\ u(x,0)=u_{0}(x)=\sum^{3}_{i=1}x^{2}_{i} & \mbox{in }\overline{D}, \end{cases} $$

where the spatial region $D= \{x=(x_{1},x_{2},x_{3}) \mid \sum^{3}_{i=1}x^{2}_{i}<1 \}$. It is easy to see that

$$p=4, \qquad h(u)=u{\mathrm{e}}^{u},\qquad g(u)=2{\mathrm{e}}^{2(u-1)}, \qquad f(u)={ \mathrm{e}}^{6u}. $$

Now we have

$$\alpha=\inf_{s\in\overline{\mathbb{R}_{+}}} \frac{f(s)}{g(s)h'(s){\mathrm{e}}^{-s}} =\frac{{\mathrm{e}}^{2}}{2}\inf _{s\in{\overline{\mathbb{R}_{+}}}} \frac{{\mathrm{e}}^{4s}}{1+s} =\frac{{\mathrm{e}}^{2}}{2} $$

and

$$\beta=\min_{\overline{D}} \frac{\nabla\cdot (|\nabla u_{0}|^{2}\nabla u_{0} )+f(u_{0})}{ g(u_{0})h'(u_{0}){\mathrm{e}}^{-u_{0}}} =\frac{{\mathrm{e}}^{2}}{2} \min _{\overline{D}} \frac{40u_{0}+{\mathrm{e}}^{6u_{0}}}{(1+u_{0}){\mathrm{e}}^{2u_{0}}} =\frac{{\mathrm{e}}^{2}}{2} \min _{0\leq s\leq1} \frac{40s+{\mathrm{e}}^{6s}}{(1+s){\mathrm{e}}^{2s}} =\frac{{\mathrm{e}}^{2}}{2}. $$

We easily verify that the three assumptions (2.5)–(2.7) of Theorem 2.1 hold. It follows from Theorem 2.1 that *u* must blow up in a finite time $t^{*}$ and

$$\begin{aligned}& t^{*}\leq\frac{1}{\beta} \int^{+\infty}_{M_{0}}\frac{{\mathrm{e}}^{\tau }}{g(\tau)}\, {\mathrm{d}}\tau = \int^{+\infty}_{1}\frac{1}{{\mathrm{e}}^{\tau}}\, {\mathrm{d}}\tau = \frac{1}{\mathrm{e}}, \\& u(x,t)\leq\Phi^{-1} \bigl(\beta \bigl(t^{*}-t \bigr) \bigr) = \Phi^{-1} \biggl(\frac{{\mathrm{e}}^{2}}{2} \bigl(t^{*}-t \bigr) \biggr) =\ln \frac{1}{t^{*}-t}. \end{aligned}$$

### Example 4.2

Let *u* be a nonnegative classical solution of the following problem:

$$\textstyle\begin{cases} (u{\mathrm{e}}^{u} )_{t}=\nabla\cdot (|\nabla u|^{2}\nabla u ) +{\mathrm{e}}^{-4u} &\mbox{in }D\times(0,T), \\ \frac{\partial u}{\partial n}=2{\mathrm{e}}^{2(1-u)} &\mbox{on }\partial D\times(0,T), \\ u(x,0)=\sum^{3}_{i=1}x^{2}_{i} & \mbox{in }\overline{D}, \end{cases} $$

where the spatial region $D= \{x=(x_{1},x_{2},x_{3}) \mid \sum^{3}_{i=1}x^{2}_{i}<1 \}$. Now

$$p=4,\qquad h(u)=u{\mathrm{e}}^{u},\qquad g(u)=2{\mathrm{e}}^{2(1-u)}, \qquad f(u)={ \mathrm{e}}^{-4u}. $$

We have

$$\xi=\sup_{s\in\overline{\mathbb{R}_{+}}} \frac{f(s)}{g(s)h'(s){\mathrm{e}}^{s}} =\frac{1}{2{\mathrm{e}}^{2}}\sup _{s\in{\overline{\mathbb{R}_{+}}}} \frac{1}{(1+s){\mathrm{e}}^{4s}} =\frac{1}{2{\mathrm{e}}^{2}} $$

and

$$\begin{aligned} \eta =&\max_{\overline{D}} \frac{\nabla\cdot (|\nabla u_{0}|^{2}\nabla u_{0} )+f(u_{0})}{ g(u_{0})h'(u_{0}){\mathrm{e}}^{u_{0}}} =\frac{1}{2{\mathrm{e}}^{2}} \max _{\overline{D}} \frac{40u_{0}+{\mathrm{e}}^{-4u_{0}}}{1+u_{0}} \\ =&\frac{1}{2{\mathrm{e}}^{2}} \max _{0\leq s\leq1} \frac{40s+{\mathrm{e}}^{-4s}}{1+s} =\frac{10}{{\mathrm{e}}^{2}}+ \frac{1}{4{\mathrm{e}}^{6}}. \end{aligned}$$

It is easy to check that the three assumptions (3.5)–(3.7) of Theorem 3.1 hold. Theorem 3.1 ensures that *u* must be a global solution and

$$u(x,t)\leq\Psi^{-1} \bigl(\eta t+\Psi \bigl(u_{0}(x) \bigr) \bigr) =\ln \Biggl[ \biggl(20+\frac{1}{2{\mathrm{e}}^{4}} \biggr)t+\exp \Biggl(\sum ^{3}_{i=1}x^{2}_{i} \Biggr) \Biggr]. $$

## Conclusion

In this paper, we research the blow-up and global solutions of *p*-Laplacian parabolic problem (1.1). We find that it is difficult to study the existence of blow-up and global solutions of problem (1.1) by using the differential inequality technique in [1]. The main reason for this is that the boundary conditions in problems (1.1) and (1.2) are different. As in [16] and [22], we combine the parabolic maximum principle with differential inequality to study problem (1.1). The difficulty of using this method is the need to construct some appropriate auxiliary functions. Since the principal parts of the two equations are different in problems (1.1) and (1.3), the auxiliary functions in papers [16] and [20] are not suitable for problem (1.1). Therefore, the key to our study is to construct new auxiliary functions *P*, Φ, *Q*, and Ψ defined in (2.3), (2.4), (3.3), and (3.4), respectively. Using these auxiliary functions, the parabolic maximum principle, and the differential inequality technique, we complete the study of (1.1). We set up the conditions on functions *f*, *g*, *h*, and $u_{0}$ to ensure that the solution of (1.1) either blows up or exists globally. In addition, an upper estimate of the global solution and the blow-up rate are obtained. We also give an upper bound for the blow-up time.

## References

[1] Ding J.T., Shen X.H. (2016). Blow-up in *p*-Laplacian heat equations with nonlinear boundary conditions. Z. Angew. Math. Phys..

[2] Kbiri Alaoui M., Messaoudi S.A., Khenous H.B. (2014). A blow-up result for nonlinear generalized heat equation. Comput. Math. Appl..

[3] Li F.S., Li J.L. (2014). Global existence and blow-up phenomena for *p*-Laplacian heat equation with inhomogeneous Neumann boundary conditions. Bound. Value Probl..

[4] Niculescu C.P., Roventa L. (2012). Generalized convexity and the existence of finite time blow-up solutions for an evolutionary problem. Nonlinear Anal. TMA.

[5] Zhang Z.C., Li Z.J. (2012). A universal bound for radial solutions of the quasilinear parabolic equation with *p*-Laplace operator. J. Math. Anal. Appl..

[6] Payne L.E., Philippin G.A., Vernier Piro S. (2010). Blow-up phenomena for a semilinear heat equation with nonlinear boundary condition, II. Nonlinear Anal. TMA.

[7] Liang Z.L., Zhao J.N. (2009). Localization for the evolution *p*-Laplacian equation with strongly nonlinear source term. J. Differ. Equ..

[8] Tersenov A.S., Tersenov A.S. (2008). The problem of Dirichlet for evolution one-dimensional *p*-Laplacian with nonlinear source. J. Math. Anal. Appl..

[9] Zeng X.Z. (2007). Blow-up results and global existence of positive solutions for the inhomogeneous evolution *p*-Laplacian equations. Nonlinear Anal. TMA.

[10] Li F.C., Xie C.H. (2003). Global and blow-up solutions to a *p*-Laplacian equation with nonlocal source. Comput. Math. Appl..

[11] Chen C.S., Wang R.Y. (2002). $L^{\infty}$ estimates of solution for the evolution *m*-Laplacian equation with initial value in $L_{q} (\Omega)$. Nonlinear Anal. TMA.

[12] Friedman A. (1964). Partial Differential Equation of Parabolic Type.

[13] Ma L.W., Fang Z.B. (2016). Blow-up analysis for a reaction–diffusion equation with weighted nonlocal inner absorptions under nonlinear boundary flux. Nonlinear Anal., Real World Appl..

[14] Ding J.T. (2016). Blow-up phenomena for nonlinear reaction–diffusion equations under nonlinear boundary conditions. J. Funct. Spaces.

[15] Harada J. (2015). Blow-up behavior of solutions to the heat equation with nonlinear boundary conditions. Adv. Differ. Equ..

[16] Zhang L.L., Zhang N., Li L.X. (2014). Blow-up solutions and global existence for a kind of quasilinear reaction–diffusion equations. Z. Anal. Anwend..

[17] Baghaei K., Hesaaraki M. (2013). Lower bounds for the blow-up time in the higher-dimensional nonlinear divergence form parabolic equations. C. R. Math. Acad. Sci. Paris.

[18] Harada J., Mihara K. (2012). Blow-up rate for radially symmetric solutions of some parabolic equations with nonlinear boundary conditions. J. Differ. Equ..

[19] Xiang Z.Y., Wang Y., Yang H.Z. (2011). Global existence and nonexistence for degenerate parabolic equations with nonlinear boundary flux. Comput. Math. Appl..

[20] Ding J.T., Guo B.Z. (2011). Global existence and blow-up solutions for quasilinear reaction-diffusion equations with a gradient term. Appl. Math. Lett..

[21] Payne L.E., Philippin G.A., Vernier Piro S. (2010). Blow-up phenomena for a semilinear heat equation with nonlinear boundary condition, I. Z. Angew. Math. Phys..

[22] Zhang H.L. (2008). Blow-up solutions and global solutions for nonlinear parabolic problems. Nonlinear Anal. TMA.

[23] Quittner P., Rodríguez-Bernal A. (2005). Complete and energy blow-up in parabolic problems with nonlinear boundary conditions. Nonlinear Anal. TMA.

[24] Chen W.Y. (2004). The blow-up estimate for heat equations with non-linear boundary conditions. Appl. Math. Comput..

[25] Fila M., Guo J.S. (2002). Complete blow-up and incomplete quenching for the heat equation with a nonlinear boundary condition. Nonlinear Anal. TMA.

[26] Rodriguez-Bernal A., Tajdine A. (2000). Dynamics of reaction diffusion equations under nonlinear boundary conditions. C. R. Acad. Sci. Paris, Sér. I Math..

[27] Protter M.H., Weinberger H.F. (1967). Maximum Principles in Differential Equations.

